# Adipose stem cells-derived exosomes modified gelatin sponge promotes bone regeneration

**DOI:** 10.3389/fbioe.2023.1096390

**Published:** 2023-02-09

**Authors:** Gen Li, Yin Zhang, Jiezhou Wu, Renhao Yang, Qi Sun, Yidong Xu, Bo Wang, Ming Cai, Yang Xu, Chengyu Zhuang, Lei Wang

**Affiliations:** ^1^ Department of Orthopaedics, Ruijin Hospital, Shanghai Jiaotong University School of Medicine, Shanghai, China; ^2^ Department of Orthopaedics, Shanghai Tenth People’s Hospital, Tongji University School of Medicine, Shanghai, China

**Keywords:** adipose stem cells, exosomes, tissue engineering, bone defect, Biomaterials

## Abstract

**Background:** Large bone defects resulting from trauma and diseases still a great challenge for the surgeons. Exosomes modified tissue engineering scaffolds are one of the promising cell-free approach for repairing the defects. Despite extensive knowledge of the variety kinds of exosomes promote tissue regeneration, little is known of the effect and mechanism for the adipose stem cells-derived exosomes (ADSCs-Exos) on bone defect repair. This study aimed to explore whether ADSCs-Exos and ADSCs-Exos modified tissue engineering scaffold promotes bone defects repair.

**Material/Methods:** ADSCs-Exos were isolated and identified by transmission electron microscopy nanoparticle tracking analysis, and western blot. Rat bone marrow mesenchymal stem cells (BMSCs) were exposed to ADSCs-Exos. The CCK-8 assay, scratch wound assay, alkaline phosphatase activity assay, and alizarin red staining were used to evaluate the proliferation, migration, and osteogenic differentiation of BMSCs. Subsequently, a bio-scaffold, ADSCs-Exos modified gelatin sponge/polydopamine scaffold (GS-PDA-Exos), were prepared. After characterized by scanning electron microscopy and exosomes release assay, the repair effect of the GS-PDA-Exos scaffold on BMSCs and bone defects was evaluated *in vitro* and *in vivo*.

**Results:** The diameter of ADSCs-exos is around 122.1 nm and high expressed exosome-specific markers CD9 and CD63. ADSCs-Exos promote the proliferation migration and osteogenic differentiation of BMSCs. ADSCs-Exos was combined with gelatin sponge by polydopamine (PDA)coating and released slowly. After exposed to the GS-PDA-Exos scaffold, BMSCs have more calcium nodules with osteoinductive medium and higher expression the mRNA of osteogenic related genes compared with other groups. The quantitative analysis of all micro-CT parameters showed that GS-PDA-Exos scaffold promote new bone formed in the femur defect model *in vivo* and confirmed by histological analysis.

**Conclusion:** This study demonstrates the repair efficacy of ADSCs-Exos in bone defects, ADSCs-Exos modified scaffold showing a huge potential in the treatment of large bone defects.

## Introduction

Despite the great advance of bone tissue engineering in the last few years, bone defect caused by various reasons such as trauma and infection is still a challenge for clinicians in orthopedic surgery (NIGUEZ SEVILLA et al., 2019; TOROS and OZAKSAR, 2019).Traditional methods to repair the bone defect include autografts, allografts, and xenografts, which exhibit excellent osteoconductive and osteoinductive properties, they still have several limits include repetitive surgery, immune rejection, and risk of disease transmission. Bioactive implants may provide an effective solution for this problem. To improve the bioactivity of the implants, many researchers use different substances, including polymer materials (DONG et al., 2015), ions (FERRANDEZ-MONTERO et al., 2019), growth factors (LIU et al., 2017; CABALLERO AGUILAR et al., 2019), cells (LIU et al., 2017), to modified different implant materials.

Among these, mesenchymal stem cells (MSCs) show strong osteogenesis activity in bone defect repair and have emerged as an alternative modified method to improve the implants (ANNAMALAI et al., 2019). However, MSCs have relative limitations in the maintenance of biological activity, and the logistics delivery in clinical therapies (AL-MORAISSI et al., 2019).

Recently, MSC paracrine effects are considered to be principally responsible for the tissue repair potential, and increasing interest has focussed on the exosomes (AGHAJANI NARGESI et al., 2017; PHINNEY and PITTENGER, 2017). MSCs derived exosomes show lower immunogenicity, and will not directly form tumors compared with MSCs. Using MSCs derived exosomes may get around MSCs’ side effects (QI et al., 2016). Compared with stem cells, MSCs derived exosomes show a safe, low-cost and efficient manner without immune or ethical restrictions (LIU et al., 2021)

Exosomes, secreted by most cell types with a diameter of 30–180 nm, are released into the extracellular environment with the cell membrane. Exosomes can carry many bioactive molecules such as lipids, proteins, mRNAs, tRNA, lncRNAs, and miRNAs (SOKOLOVA et al., 2011; GURUNATHAN et al., 2019). Researchers found the source of MSCs influence the biological effects of MSC-derived exosomes. MSCs obtained from bone marrow (BMSCs) are the most frequently used stem cells in cell therapy and tissue engineering. BMSCs derived exosomes also promote bone regeneration (FURUTA et al., 2016). Although most research on MSC-based cell therapy focused on BMSCs, there is an increasing importance of adipose tissue as an alternative MSCs source. Compared with an equivalent amount of bone marrow, adipose tissue can provide up to 500-fold more MSCs (HASS et al., 2011). Thus, adipose tissue can be the most efficient source of MSCs (DUSCHER et al., 2017). Several studies show that exosomes derived from adipose-derived stem cells (ADSCs-Exos) promote angiogenesis, cutaneous healing, and nerve regeneration (YANG et al., 2018; HONG et al., 2019). However, the repair effect of ADSCs-Exos on bone defect remains unclear. Here, we aim to evaluate the impact of ADSCs-Exos on BMSCs osteogenic differentiation *in vitro* and repair effect of ADSCs-Exos after combined with materials on bone defect *in vivo*, to estimate its potential therapeutic value on bone defect.

## Materials and methods

### Ethics statement and animals

All the animal procedures and operations complied with the Guidelines of Shanghai Laboratory Animal Center and the Policies on the Use of Humans and Animals in Research of the Shanghai Tenth People’s Hospital (SYXK: 2014-0026) and demonstrated to the principles outlined in the Declaration of Helsinki. All the animals in this work were looked after humanely, and all efforts were made to remove their discomfort.6-weeks and 8-weeks age male Sprague–Dawley (SD) rats were purchased from Bikai Experimental Animal Company (Shanghai, China). All the rats were housed in the standard cage with thermostatic room (25°C) in a 12 h light and 12 h dark cycle. The standard diet and water were supplied. The rats were adapted to the experimental environment at least 2 weeks before starting this study.

### Isolation and culture of ADSCs

ADSCs derived from the inguinal fat pad of 6-weeks SD rats. In brief, the capillaries were removed from the lipoaspirate, minced with phosphate-buffered solution (PBS, GenomSciences, Hangzhou, China) and digested with 1% collagenase A (Gibco, Carlsbad, United States) for 1 h at 37°C. After filtration, the mixture was centrifuged (1,000 rpm, 10 min) at room temperature (RT), and the supernatant was discarded. The collected cells were washed with PBS, centrifuged (1,000 rpm, 5 min) and then resuspended in DMEM/F12 medium (Gibco, Carlsbad, United States) with 10% fetal calf serum (FCS; Gibco, Carlsbad, United States) and 1% penicillin-streptomycin (PS) in a humidified 5% CO_2_ at 37°C; When the cells passed 3 generations, they were identified and used for next experiments.

### Flow cytometry

This study used stem cell surface markers to evaluate the phenotype of cells, including CD29-PE, CD90-FITC, CD105-PE-cy5, CD45-APC (all the antibodies from Abcam, Cambridge, United Kingdom) after 1 week expansion. In brief, cells were cultured with antibodies for 30 min at room temperature, and the analysis was performed by a FACSCalibur flow cytometer (BD Biosciences, San Jose, United States). The nonspecific IgG-labeled cells were used as controls. The data were analyzed by FlowJo software (Tree Star, Ashland, OR).

### ADSCs differentiation

ADSCs (2*10^5^ per well in 6-well plates) were treated with adipogenic media to induce adipogenesis. Adipogenic medium consisted of 0.5 mM 3-isobutyl-1-methyl-xanthine, 1 μM dexamethasone, 5 μg/mL insulin, and 50 μM indomethacin. The culture medium was replaced every 3 days. Seven days later, Oil Red O staining (Beyotime Institute of Biotechnology, Haimen, China) was used to measure lipid vesicles. For osteogenic differentiation, Osteogenic medium consisted of 100 nM dexamethasone, 10 mM βglycerophosphate, and 0.05 mM L-ascorbic acid-2-phosphate, the media was changed with osteogenic induction media supplied with the differentiation media every 3 days. After 21 days, osteonectin was detected. For chondrogenic differentiation, ADSCs were cultured in a chondrogenic differentiation medium (Cyagen, Santa Clara, CA, United States) and refreshed every 3 days. 21 days later, cartilage matrix protein was detected. All the pictures were captured by an Olympus IX51 light microscope (Olympus, Tokyo, Japan).

### Exosomes isolation and identification

The exosomes derived from ADSCs were purified from serum-free cell medium by series centrifugation and filtration steps. Briefly, collected supernatant centrifuged at 300 g for 10 min to remove dead cells, 5,000 g for 10 min to remove cellular debris. Then the supernatant was ultrafiltrated by 0.22 μm filter (Whatman, Maidstone, United Kingdom) and centrifuged at 120,000 g for 90 min. The final pellets were resuspended in 1 mL PBS and stored at −80°C.The collected pellets were distinguished by transmission electron microscopy (TEM, Zeiss, Axio, Germany) and nanoparticle tracking analysis (NTA, NanoSight LM10, Malvern Instruments, Westborough, MA).

### PKH67-labeled exosomes

ADSCs derived exosomes were labeled with PKH67 (Sigma-Aldrich, St. Louis, MO). In brief, 2 μL PKH67 was incubated with exosomes in a total of 1 mL of diluent for 15 min. 1 mL of 1% of BSA was added into the mixture to stop the labeling and was centrifuged at 120,000g for 2 h in 4°C. Then, the supernatant was discarded, resuspended the pellets in 5 mL of PBS, and centrifuged at 120,000g for 1 h in 4°C. Finally, PKH67-labeled-exosomes were resuspended in 1 mL of PBS and stored at −80°C.

### Western blot

ADSCs or exosomes were lysed in RIPA buffer (Beyotime, Shanghai, China). The samples were separated on a 10%–15% SDS polyacrylamide gel and transferred to polyvinylidene difluoride (PVDF, Millipore, Billerica, MA, US) membranes. Then the PVDF membranes were blotted with 5% BSA for 1 h and incubated with primary antibodies overnight at 4°C. The membranes were incubated with horseradish peroxidase-conjugated anti-rat IgG (1:5,000, Santa Cruz Biotechnology, US) and visualized with an enhanced chemiluminescence kit (Amersham, GE Healthcare, Waukesha, WI, US) and quantified by ImageJ software ver. 1.52. Primary polyclonal antibodies against CD9 and CD63 (1: 1,000, Cell Signaling Technology).

### Fabrication and characterization of adipose stem cells-derived exosomes modified gelatin sponge/polydopamine scaffolds (GS-PDA-Exos)

Gelatin sponges were purchased from Guangzhou Kuaikang Medical Apparatus Co. (Guangzhou, China). According to the previous study (DINH et al., 2018), the gelatin sponges soaked in dopamine (DA) solution (2 mg/mL in 10 mM Tris-HCl, pH 8.5, Sigma-Aldrich, St. Louis, US) were incubated with shaking at 37°C for 24 h to form the polydopamine (PDA) coating. To remove the non-adherent PDA, the gelatin sponge/polydopamine (GS-PDA) scaffolds were gently shaken in an ultrasonic cleaner with distilled water for 5 times. The GS-PDA scaffolds were dried and sterilized by ethylene oxide before the next step. Then the GS-PDA scaffolds were immersed in PKH67-labeled or non-labeled ADSCs-Exos solution (10^10^particles/scaffold) with shaking at 37°C for 12 h. To remove the non-adherent exosomes, and the GS-PDA-Exos scaffolds were gently shaken in an ultrasonic cleaner with distilled water for 5 times. The distribution of PKH67-labeled exosomes on the scaffolds was observed with the confocal imaging system (Nikon, Japan). To measure the ADSCs-Exos release effect of GS-PDA-Exos scaffolds. The amount of ADSCs-Exos released was measured using CD63 ELISA (Beyotime, Shanghai, China) assay. Scanning electron microscopy (SEM, Hitachi, Tokyo, Japan) was used to observe the surface morphology of the scaffolds.

### Isolation and culture of BMSCs

BMSCs were isolated from the femurs and tibias of 6-week SD male rats. In brief, The bone marrow was separated from the femurs and tibias of rats by flushing with a serum-free culture medium (DMEM/F12; Gibco, USA). The red blood cells were lysed in the lysate and cultured in DMEM/F12 (containing 10% FBS) and 1% penicillin-streptomycin (PS) in a humidified 5% CO2 at 37°C. Change the medium after 24–48 h, then replace the medium once every 2–3 days later. When the cells passed 3 generations, they were identified and used for next experiments BMSCs used in this study were P3-P6 generation cells.

### Cell proliferation and migration assay

The regulation of ADSCs-Exos and GS-PDA-Exos on the proliferation of rat BMSCs was assessed by the Cell Counting Kit-8 (CCK-8) assay (Dojindo, Kyushu Island, Japan) (ZHU et al., 2019). Briefly, BMSCs were seeded into 96 well plates at a density of 4,000 cells per well, respectively. The cells were cultured with 100 μL of growth medium containing different concentrations of exosomes (0, EXO-L 1 × 10^9^ particles/mL, EXO-H 1 × 10^10^ particles/mL) or 100 μL of growth medium with scaffolds immersed. Each well was incubated with 10 μL CCK-8 solution and cell proliferation curves were constructed at a wavelength of 450 nm.

Scratch wound assays assessed the effect of ADSCs-Exos and GS-PDA-Exos on Cell migration. 1.5 × 10^5^ BMSCs were seeded into 6-well plates and cultured in growth medium for 12 h. Next, one scratch was made in each well using a 200 μL pipette tip. After washing with PBS, the medium was then replaced with serum-free medium supplemented with ADSCs-Exos (0, 1 × 10^9^, 1 × 10^10^ particles/mL), or serum-free medium with scaffolds immersed. Wound closure was measured by capturing images at different time points. The scratched areas were measured using Image-Pro Plus software.

### Alkaline phosphatase activity assay and alizarin red staining

As described above, BMSCs were seeded into 6 well plates and cultured with growth medium containing different concentrations of exosomes or with scaffolds immersed. After 24 h, the medium was replaced with the normal or osteoinductive (OIC) medium for 10 days, At different time points (1, 4, 7, and 10 days), the alkaline phosphatase (ALP) activity assay of the supernatant was performed with an ALP Assay Kit (Beyotime, Shanghai, China). To assess mineralization, cells were induced for 3 weeks, fixed with 4% paraformaldehyde solution and washed with PBS for 3 times, then gently washed with distilled water and stained with 2% Alizarin red (ARS, Sigma-Aldrich, St. Louis, US). To quantify the coloration of ARS. 10% of acetic acid was added to each cell. After incubation for 12 h, the suspension transferred to tubes and centrifuged at 20,000 g for 15 min. The supernatant was transferred to another tube and neutralized with 10% ammonium hydroxide. Then 100 μL of each sample was measured at a wavelength of 405 nm.

### RNA extraction and real-time quantitative-PCR (RT-PCR)

RT-PCR assessed the regulation of ADSCs-Exos and GS-PDA-Exos on the osteogenic differentiation related genes. Rat BMSCs were grown in normal, and osteoinductive media in the presence of ADSCs-Exos or scaffolds. The total RNA of the samples was isolated based on the manufacturer’s protocol using the RNAiso plus kit (TaKaRa, Tokyo, Japan). The RNA was treated with DNase in a 10 µL reaction and conducted at 42°C for 2 min. Then, the PrimeScript RT reagent Kit (TaKaRa, Tokyo, Japan) was used in a total volume of 20 µL for the mRNAs in a 15 min incubation period at 37°C and stopped after a 5 s enzyme denaturing step at 85°C.

Real-time amplification was performed by the SYBR Premix Ex Taq (TaKaRa, Tokyo, Japan) in an ABI 7900 thermocycler (ABI; Foster City, CA, United States). The PCR cycling was 95°C for 30 s, followed by 40 cycles of 95°C for 5 s and 60°C for 30 s. Then a dissociation curve was at 95°C for 15 s, 60°C for 15 s, and 95°C for 15 s. GAPDH was used as a relative control and analyzed using the 2 the number of ^ -ΔΔCT method. Primer sequences used in this work were described in Supplementary Table S1.

### Animal experiment

The studies involving animals were reviewed and approved by RuiJin Hospital Ethics Committee. Eight-week-old male Sprague Dawley (SD) rats were obtained from the Bikai Experimental Animal Company (Shanghai, China). In total, 24 animals were randomly divided into four groups as follows: 1) empty defect (control) 2) GS scaffold 3) GS-PDA scaffold and 4) GS-PDA-Exos scaffold, each group has 6 animals. After adaptation for two weeks, 300∼350 g SD rats were used for establishing the critical-sized rat femoral bone defect model. Briefly, All animals were anesthetized by intraperitoneal injecting 2.5% pentobarbital (40 mg/kg). The knee was shaved and sterilized, after cut open one by one to expose the distal femur, a diameter of 3.0 mm defect was made at the femoral condyle with a slow-speed electric drill. The ice saline solution was locally used to lower the temperature. Then the scaffolds were implanted into the defects, and the incision was stitched.

### Radiologic and histological analysis

At 2 and 4 weeks after implantation, the femurs of the animals were harvested and evaluated the new bone formation within the bone defect using micro-computed tomography (micro-CT; Skyscan 1,076, Bruker, Belgium) with a spatial resolution of 12 μm. The center of the bone tunnel of a 3 mm diameter was selected as the region of interest (ROI). The Scanco software was used to measure bone mineral density (BMD), bone tissue volume/total tissue volume (BV/TV), trabecular thickness (Tb.Th) and trabecular separation/spacing (Tb.Sp). After the micro-CT scan, the femurs were used for histology analysis. The samples were fixed in 4% paraformaldehyde for 48 h and decalcified in 10% ethylenediaminetetraacetic acid for 4 weeks. After decalcification, the samples were dehydrated and embedded in paraffin. Hematoxylin and eosin (HE) staining was performed with the 5 μm thick sections.

### Statistical analysis

The SPSS 17.0 software was used for statistical analyses. All data were presented as mean ± standard deviation (SD). Differences among groups were assessed with one-way ANOVA. The statistical significance was *p* < 0.05. All experiments were repeated at 3 replicates.

## Results

### Acquisition and identification of ADSCs in SD rats

**FIGURE 1:**
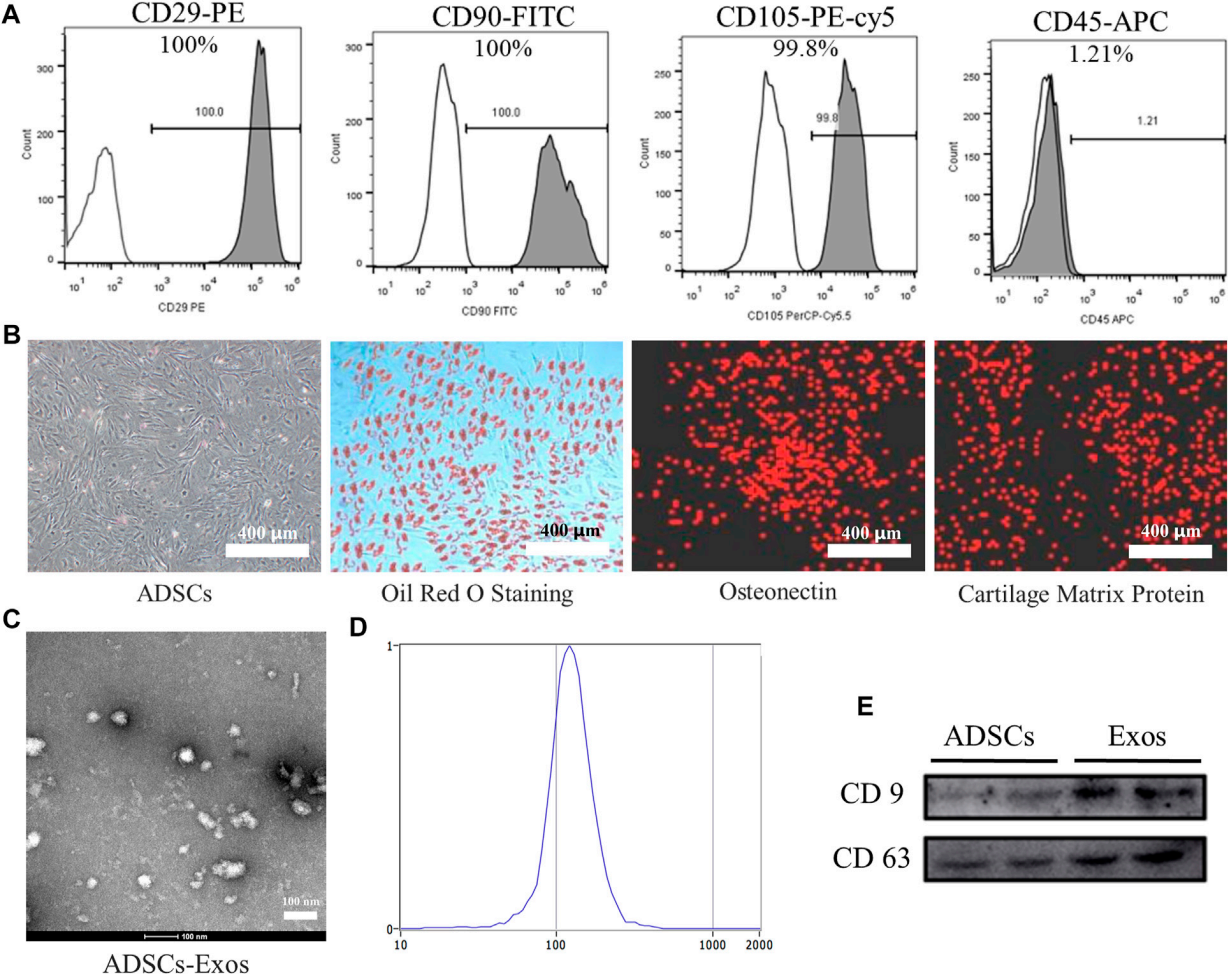
Identification of ADSCs and ADSCs-Exos. **(A)**. Characterization of ADSCs marker expression by flow cytometry analysis. ADSCs stained with CD29-PE, CD90-FITC, CD105-PE-cy5, and CD45-APC antibodies, respectively. **(B)**. Undifferentiated and differentiated ADSCs. Phenotypic characterization of ADSCs at high confluency; ADSCs were induced to differentiate into adipogenic cells under adipogenic differentiation conditions for 21 days and stained positive for Oil Red O; Under osteogenic differentiation conditions, ADSCs formed Calcium crystals and expressed osteonectin by immunofluorescence stained; Under chondrogenic differentiation conditions, ADSCs differentiate into chondrogenic cells and immunohistochemical stained positive for cartilage matrix protein. (200×) **(C)**. Morphology of exosomes derived from ADSCs (ADSCs-Exos) observed by transmission electron microscopy (TEM). **(D)**. Particle size distribution of ADSCs-Exos measured by nanoparticle tracking analysis: the diameter of exosomes mainly distributed at 80–180 nm; the peak value is 122.1 nm. **(E)**. Western blots of exosomal protein of ADSCs-Exos. Blots were probed using antibodies against CD9 and CD63.

We obtained the ADSCs by standard methods and then performed the identification of surface markers. Flow cytometry results showed that over 99% of cells (passage 1) were positive for the mesenchymal stem cell markers CD29, CD90, or CD105 and negative for the leukocyte marker CD45 (Figure 1A). The ADSCs underwent 21-day chondrogenic, and adipogenic induction and experienced 14-day osteogenic induction to evaluate their multilineage differentiation potential. The ADSCs were positive for Oil Red O staining after adipogenic induction (Figure 1B). Cell immunofluorescence showed the ADSCs cultured in osteogenic induction positive expression of osteonectin. After adipogenic induction, ADSCs showed the expression of cartilage matrix protein as detected by immunofluorescence. The results indicate that the cells we obtained are ADSCs (Figure 1B).

### Characterization of ADSCs-derived exosomes

TEM analysis showed that the exosomes isolated from the supernatants of ADSCs is a round membranous vesicle, which diameter of approximately 100 nm (Figure 1C).NTA analysis indicated that the parameters of the exosomes ranged mainly from 80 to 180 nm (Figure 1D). Western blotting analysis (Figure 1E) showed that the exosome-specific markers CD63 and CD9 were detected in ADSCs-derived exosomes, and the expression of abundance is high.

### ADSCs-derived exosomes promote BMSCS proliferation, migration, and osteogenic differentiation

Recent research has found that exosomes are the key substance of the paracrine function of stem cells for participating in the repair process of various tissues. Therefore, exosomes may be an ideal material to improve the osteogenic differentiation ability of stem cells.

**FIGURE 2:**
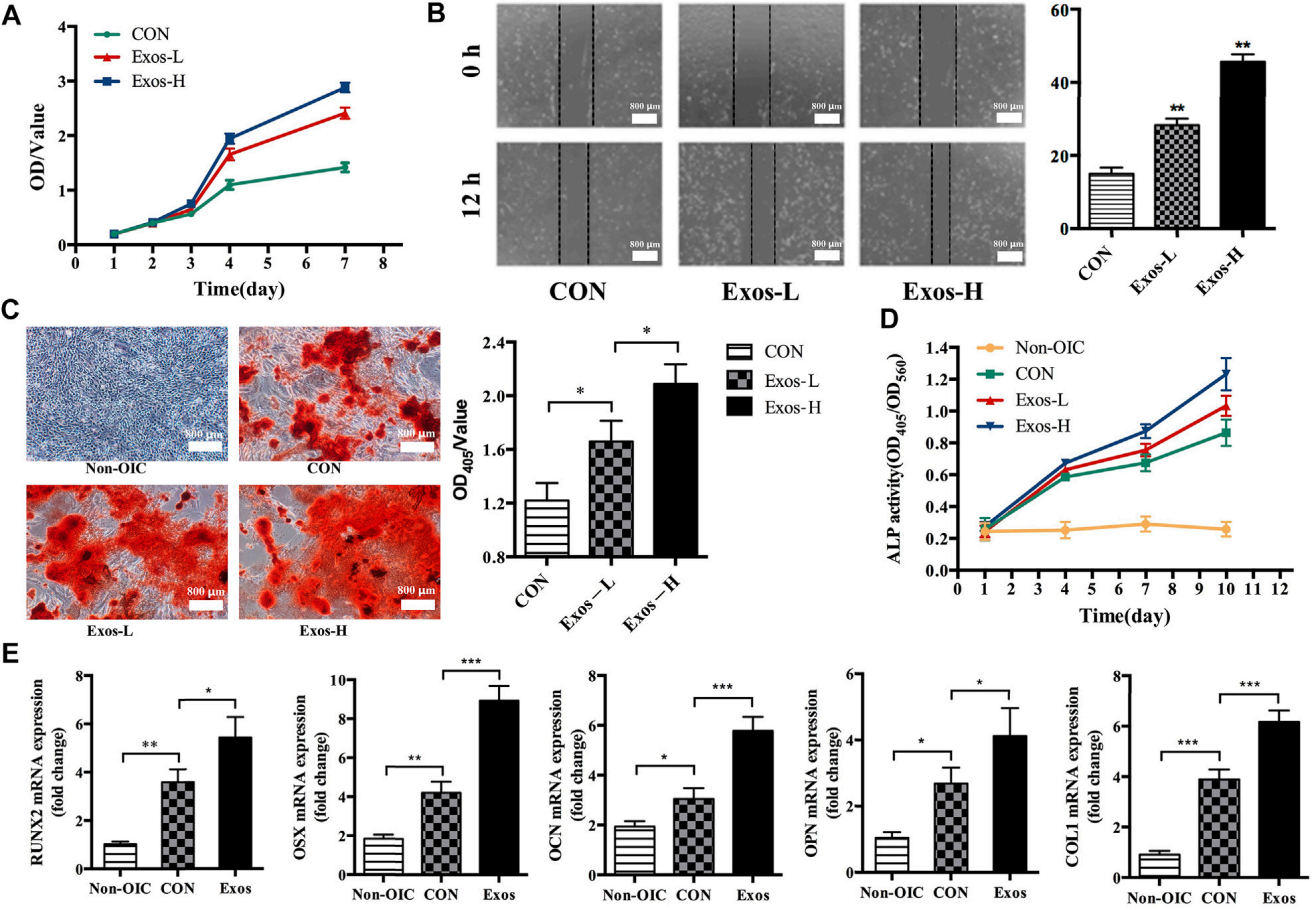
ADSCs-Exos promote the proliferation, migration, and osteogenic differentiation of BMSCs. **(A)**. CCK-8 assay showed that BMSCs treated by Exos-H (1 × 10^10^ particles/ml) cultured grows faster with culture days as compared to Exos-L (1 × 10^9^ particles/ml) or CON in conditioned-media. **(B)**. The scratch wound assay of BMSCs treated with Exos-H, Exos-L, and CON, respectively. By 12 h, the scratch test results showed that compared with the CON group, BMSCs had longer migration distances in both Exos-H and Exos-L groups. **(C)**. Microscopic images showing the formation of mineralized nodules. The number of BMSCs in the normal medium (NON-OIC) group increased gradually within 21 days, but no mineralized nodules observed. On day 21, mineralized nodules were found in CON, Exos-L, Exos-H groups by ARS staining. Among them, the number of mineralized nodules in Exos-L, Exos-H group is larger than that in CON, and the Exos-H group has the most mineralized nodules. **(D)**. The activity of alkaline phosphatase (ALP) in BMSCs was detected at 1, 4, 7, and 10 days. The ALP activity of Exos-L and Exos-H group was significantly higher than that of the CON group at the time points of 7 and 10 days, and there was a significant difference between the groups of Exos-L and Exos-H. **(E)**. The effect of ADSCs-Exos on osteogenic differentiation related genes, including RNUX2, OSX, OCN, OPN, and COL1a. BMSCs from ADSCs-Exos group cultured in OIC medium for 10 days high expressed mRNA of osteogenic differentiation related gene. (*, *p* < 0.05; **, *p* < 0.01; ***, *p* < 0.001).

The CCK-8 assay was used to determine whether ADSCs-derived exosomes affects the proliferation activity of BMSCs. Compared with the Exos-L and CON group, the OD value of Exos-H increased significantly at 4 and 7 days, and the results showed that ADSCs-derived exosomes could promote the proliferation of BMSCs (Figure 2A). After BMSCs were treated with Exos and stimulated for 12 h, the scratch test results showed that the migration distance of BMSCs cells in the Exos-H group was longer than that in the Exos-L group. The mobility of Exos-L was (28.33 ± 3.05) %. The migration rate of Exos-H was (45.67 ± 3.51) %, which was statistically significant compared with the migration rate of the CON group (15.00 ± 3.00) % (Figure 2B). The result suggests that ADSCs-derived exosomes can promote the migration of BMSCs cells. And the effect is related to the number of exosomes particles. ARS staining and quantitative analysis showed that compared with Exos-L and CON, the Exos-H group had the most significant number of mineralized nodules. And ADSCs-derived exosomes could promote the mineralization of BMSCs as the concentration increases (Figure 2C). The expression level of ALP in BMSCs treated by ADSCs-derived exosomes during osteogenesis induction was detected to evaluate whether the exosomes promoted osteogenic differentiation. The results showed that ADSCs-derived exosomes could promote BMSCs osteogenic differentiation, which is related to the exosomes concentration (Figure 2D). Further RT-PCR results showed that ADSCs-derived exosomes could increase mRNA expression of runt-related transcription factor 2 (Runx2), Osterix (OSX), osteocalcin (OCN), osteopontin (OPN), and Collagen type I (COL1) during osteogenesis induction in BMSCs (Figure 2E).

### Fabrication and characterization of GS-PDA-Exos scaffolds

**FIGURE 3:**
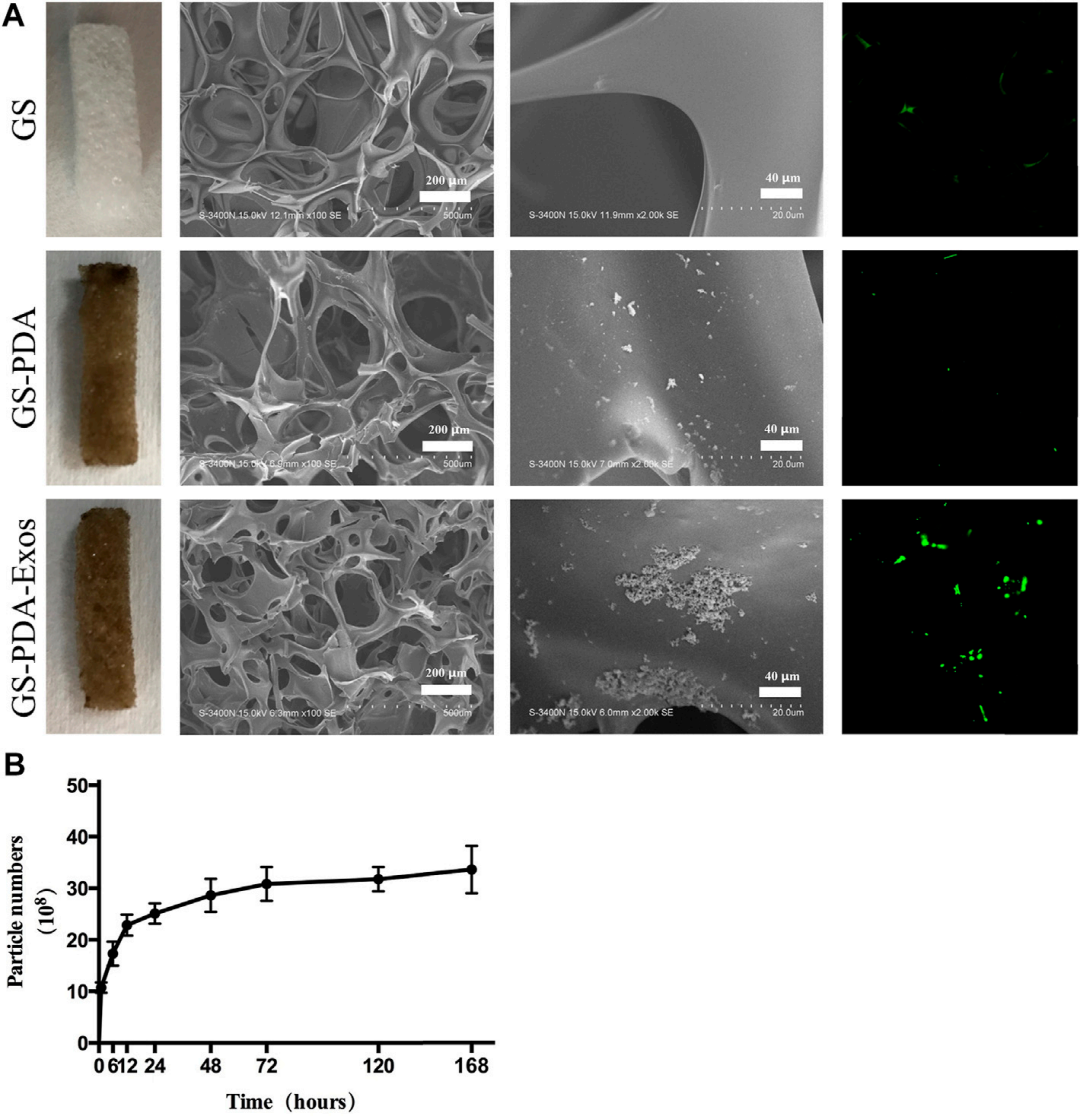
Characterization of GS-PDA-Exos scaffolds **(A)**. Three scaffolds including gelatin sponge (GS), GS with the polydopamine coating scaffold (GS-PDA), and ADSCs-Exos modified GS-PDA scaffold (GS-PDA-Exos) were prepared. Scanning electron micrographs showing the surface morphology of the scaffolds. The adhesion of PKH67 labeled ADSCs-exos on GS-PDA-Exos was observed by fluorescence microscopy. **(B)**. The release curve of ADSCs-Exos from GS-PDA-Exos scaffold.

As shown in Figure 3A, the GS turned brown or black after soaked in DA solution and formed the PDA coating, but the GS-PDA and GS-PDA-Exos scaffolds maintained their shape as naked GS. The form of the PDA coating did not cause damage to the structure of the fibrous. Shown in SEM pictures, the surface of the GS scaffold was smooth, but that of GS-PDA and GS-PDA-Exos scaffolds exhibited increased surface roughness. Similarly, A considerable number of PKH-67-labeled exosomes (green dots) could be found on the surface of the scaffolds with the confocal scanning microscopic imaging. The cumulative release curve of the amount of ADSCs-Exos released from GS-PDA-Exos scaffolds is shown in Figure 3B; the ADSCs-Exos showed burst release within 3 days, followed by a relatively slow release until 7 days.

### GS-PDA-Exos scaffold promotes osteogenesis differentiation of rat BMSCs in Vitro

**FIGURE 4:**
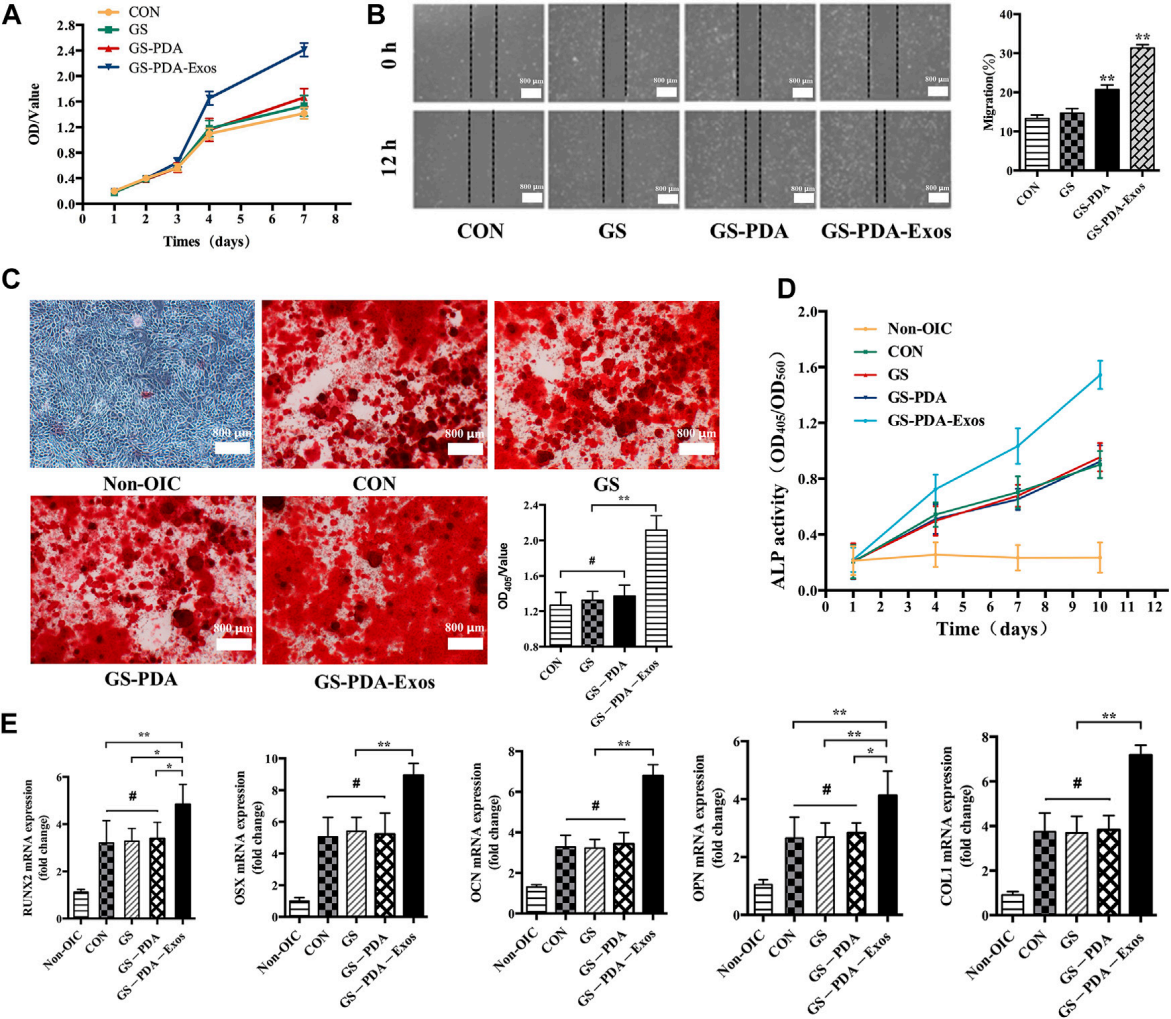
GS-PDA-Exos scaffold promotes the proliferation, migration, and osteogenic differentiation of BMSCs **(A)**. The CCK-8 assay detected the effects of GS, GS-PDA, and GS-PDA-Exos on BMSCs proliferation. **(B)**. The scratch wound assay detected the effects of GS, GS-PDA, and GS-PDA-Exos on the migration of BMSCs. Compared with the CON, GS, and GS-PDA, the mobility of BMSCs in the GS-PDA-Exos group increased significantly. **(C)**. The number of mineralized nodules of BMSCs was detected by ARS staining. The results showed a large number of mineralized nodules were observed in CON, GS, GS-PDA, and GS-PDA-Exos groups. The number of stained nodules in GS-PDA-Exos was significantly higher than that in other groups. **(D)**. The ALP activity of BMSCs was detected after exposure to the CON, GS-PDA, and GS-PDA-Exos scaffolds. The expression level of ALP in GS-PDA-Exos increased continuously within 10 days. At 4 days, it was higher than that of the CON, GS and GS-PDA groups, but there was no significant difference, while at 7 and 10 days, ALP expression was significantly higher than that of the other groups. **(E)**. Effects of materials on mRNA expression of BMSCs osteogenesis related genes (RUNX2, OSX, OCN, OPN, and Col1a) detected by RT-PCR. There was no significant difference between GS and GS-PDA and CON. Compared with other groups, GS-PDA-Exos significantly promoted the expression of RUNX2, OSX, OCN, OPN, and Col1a. (^#^, no significant difference *, *p* < 0.05; **, *p* < 0.01).

The proliferation of rat BMSCs were compared at days 1, 2, 3, 4, and 7. The cell proliferation of BMSCs increased with time within the 7 days monitoring span. Compared with GS and GS-PDA scaffolds and control groups, the optical density value in the GS-PDA-Exos scaffold group was significantly higher from the fourth to seventh day (Figure 4A). The migration ability of BMSCs with GS-PDA or GS-PDA-Exos was enhanced (Figure 4B). Alizarin Red S staining showed GS-PDA-Exos significantly increased the calcified nodules and promoted BMSCs osteogenesis (Figure 4C). Compared with other groups, the ALP activity of the GS-PDA-Exos scaffold group was higher on day 7 and 10 days (Figure 4D). After cultured in OIC media for 10 days, all osteogenic differentiation-related genes of BMSCs, including RUNX2, OSX, OCN, OPN, and Col1 significantly upregulated in GS-PDA-Exos group than that in other groups (Figure 4D). These data demonstrated the GS-PDA-Exos scaffold could promote osteogenesis *in vitro*.

### GS-PDA-Exos promotes bone repair in the femur defect model *in vivo*

**FIGURE 5:**
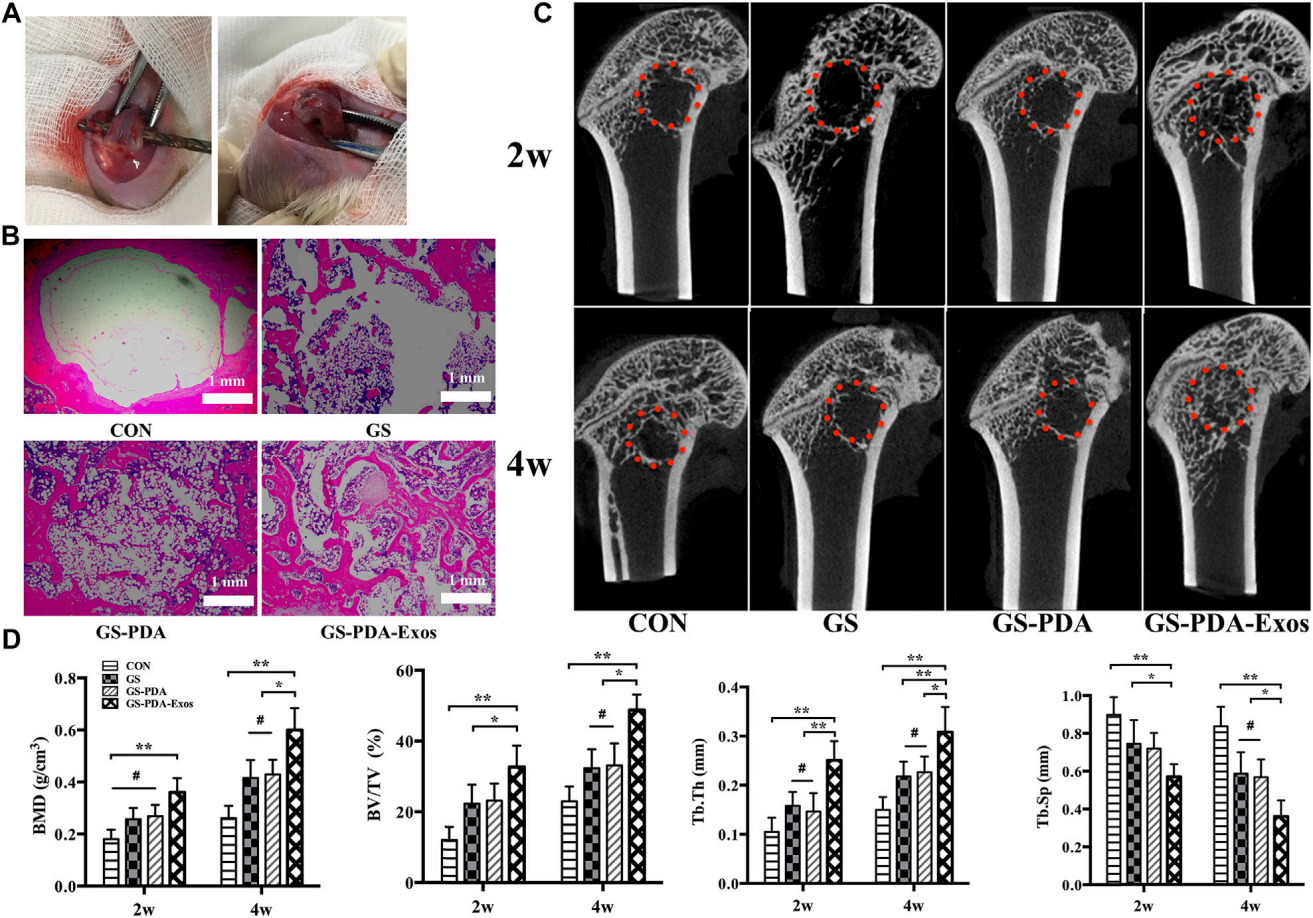
Micro-CT and histological analysis of the effect of GS-PDA-Exos on bone repair *in vivo*. **(A)**. Construction of the critical-sized femoral bone defect model of rats. **(B)**. Representative images of hematoxylin-eosin (HE) staining of the decalcification bones slice, showing the newly formed tissue, including the fibrous tissue, newly mineralized bone tissue. **(C)**. Representative two-dimensional micro-CT images showing the effect of different scaffolds (CON, GS, GS -PDA, and Gs-PDA-Exos) on the new bone tissue formation inside the defect site. The red dot line circled the region of interest (ROI). **(D)**. Summarized data showing the micro-architectural parameters of the newly formed bone tissue at 2 and 4 weeks by analyzing the micro-CT images using image analysis software. Bone mineral density (BMD), bone tissue volume/total tissue volume (BV/TV), trabecular thickness (Tb.Th), and trabecular separation/spacing (Tb.Sp) were shown in the panel. (^#^, no significant difference *, *p* < 0.05; **, *p* < 0.01).

The critical-sized femoral bone defect model of rats were made to analysis the effect of scaffolds on bone repair (Figure 5A). After 2 or 4 weeks of implantation, animals’ femurs harvested for micro-CT and histological tests. In the representative HE stained sections, the defect areas were filled with newly formed tissues in GS, GS-PDA, and GS-PDA-Exos groups. On the contrary, newly formed bone tissue was observed only along the border of the defect in the control group (Figure 5B). The bone defect area of the rat femoral defect was analyzed by micro-CT. As shown in Figure 5C, GS-PDA-Exos group had obvious new bone formation as compared with other groups at 2 and 4 weeks. The quantitative analysis of all micro-CT parameters, including BMD, BV/TV, Tb.Th and Tb. Sp confirmed that GS-PDA-Exos promotes bone repair in the femur defect model *in vivo* (Figure 5D).

## Discussion

The exosomes are released by most cell types and involved in a wide range of tissue regeneration, including the skin, heart, kidney, and skeletal musculature (DONG et al., 2019; GRANGE et al., 2019; MENDT et al., 2019). Taisuke Furuta et al. revealed that exosomes derived by bone marrow-derived MSCs promote fracture healing in a mouse model (FURUTA et al., 2016). In this study, we have successfully synthesized PDA coated GS for the controlled delivery of GS-PDA-Exos. We aimed here to elucidate whether ADSCs-derived exosomes could promote bone repair and whether GS-PDA-Exos could promote the new bone generation of a critical-sized defect in the SD rat femur. Based on the *in vitro* and *in vivo* experiments, ADSCs-derived exosomes contribute to bone repair *via* inducing BMSCs osteogenic differentiation *in vitro*, and the implant of GS-PDA-Exos exerted an excellent bone healing capability in the critical-sized bone defect of the SD rat femur.

Different kinds of MSCs have been used for tissue engineering and regenerative medicine. Among these, BMSCs serve as ideal seed cells for tissue engineering in numerous studies, ADSCs share many of the characteristics of BMSCs. Meanwhile, ADSCs can be obtained more easily *in vitro* and abundant storage *in vivo*. With varies advantages, ADSCs are more applicable than other MSCs to use for tissue engineering. As shown in Figures 1A, B, the characterization of ADSCs was identified by flow cytometry and adipogenic, osteogenic, and chondrogenic differentiation, as in previous study (CHEN et al., 2019). Emerging evidence has shown the crucial roles of exosomes from MSCs promote tissue repair (CHEW et al., 2019). MSCs-derived exosomes, as a critical product of paracrine secretion, are a kind of sphere-shaped extracellular vesicle whose diameter is about 30–180 nm. Previous studies have demonstrated that exosomes secreted by human-induced pluripotent stem cells could promote bone regeneration in critical-sized calvarial defects (QI et al., 2016). Qin et al. reported that exosomes secreted by BMSCs could improve bone regeneration in a calvarial defects model (QIN et al., 2016). Thomas Thomou et al. reported that Adipose-derived exosomal miRNAs could regulate gene expression in other tissues and serve as a vital adipokine (THOMOU et al., 2017). Based on these findings, we hypothesized that ADSCs-derived exosomes could promote bone regeneration. Firstly, the exosomes were isolated from ADSCs serum-free cell medium and characterized by TEM, NTA, and western blot. (Figures 1C–E).Secondly, Our results displayed ADSCs-derived-exos effectively accelerate the migration and proliferation of BMSCs (Figures 2A, B). More importantly, ADSCs-derived-exos could significantly increase the mRNA expression of osteogenesis-related genes such as RUNX2, OSX, OCN, OPN, COL1 (Figure 2E).Similarly, ALP activity was dramatically higher in ADSCs-derived-exos groups (Figure 2D). Compared with the control group, the ADSCs-derived-exos significantly enhanced the mineralized matrix production of BMSCs when culturing for osteogenesis differentiation (Figure 2C). Our results suggested ADSCs-derived-exos effectively promote BMSCs’ osteogenic differentiation.

To deal with bone defects, varies kinds of bone graft substitutes are being synthesized to promote bone repair (KOKUBO et al., 2004; CAO et al., 2014; KANDA et al., 2015). In this study, we use GS as a scaffold to combine ADSCs-derived-exos.GS is a partial hydrolysis product of collagen with excellent biocompatibility, biodegradability, and low immunogenicity. Besides this, high interconnected pores with macroporous morphology of the GS could enhance cell growth. These characteristics make GS an ideal biomaterial for tissue engineering. To better carry exosomes than usual physical adsorption methods, here we introduce the mussel-inspired PDA coating to adhere to and carry exosomes. The PDA coating has strong adhesion to various substrates and could second modification by other substrates through chemical reaction (LAVOIE et al., 2005). By this method, we synthesized a new biomaterial GS-PDA-Exos. As shown in Figure 3, exosomes could be immobilized by PDA coating and slowly released from the material.

Further, we investigated the effect of GS-PDA-Exos *in vitro* and *in vivo*. *In vitro*, Compared with other groups, GS-PDA-Exos could accelerate the migration and proliferation of BMSCs (Figures 4A, B), promote the activity of ALP (Figure 4D), increase mineralized nodules formation (Figure 4C), and significantly upregulated osteogenic differentiation-related genes, including, RUNX2, OSX, OCN, OPN, COL1 (Figure 4E). Based on these findings, we further investigated the *in vivo* osteogenesis functionality of the GS-PDA-Exos with a femur defect model of rats (Figure 5A). Consistent with the observation *in vitro*, micro-CT and histological analyses showed that the GS-PDA-Exos significantly promote bone repair in the femur defect model than the control groups (Figures 5B–D).

In summary, the present study suggests that ADSCs-derived exosomes enhance the migration, proliferation, and osteogenic differentiation of BMSCs *in vitro*, and a new tissue engineering GS-PDA scaffold combined with ADSCs-derived exosomes significantly enhanced bone repair in the femur defect model *in vivo*.

## Data Availability

The original contributions presented in the study are included in the article/Supplementary Material, further inquiries can be directed to the corresponding authors.
